# Primary Solid Pseudopapillary Tumor of the Ovary: A Case Report and Review of the Literature

**DOI:** 10.3390/jcm13102791

**Published:** 2024-05-09

**Authors:** Juhun Lee, Seung Ho Song, In Hee Lee, Dong Ja Kim, Hyun Jung Lee

**Affiliations:** 1 Department of Obstetrics and Gynecology, School of Medicine, Kyungpook National University, Kyungpook Nation University Hospital, Daegu 41944, Republic of Korea; gyjhlee@knu.ac.kr; 2 Department of Surgery, School of Medicine, Kyungpook National University, Kyungpook National University Hospital, Daegu 41944, Republic of Korea; jojocrom@naver.com; 3 Department of Hematology/Oncology, School of Medicine, Kyungpook National University, Kyungpook National University Chilgok Hospital, Daegu 41404, Republic of Korea; cakey83@hanmail.net; 4 Department of Forensic Medicine, School of Medicine, Kyungpook National University, Kyungpook National University Hospital, Daegu 41404, Republic of Korea; dongja222@knu.ac.kr

**Keywords:** solid pseudopapillary tumor, ovarian cancer, rare tumor, *CTNNB1*

## Abstract

Introduction: Solid pseudopapillary neoplasms (SPNs) are rare and mainly originate from the pancreas. SPNs originating from the ovary (SPN-O) are extremely rare, and only 13 cases have been reported in the English literature since 2010. Case: We report a 31-year-old woman with SPN-O accompanied by multiple metastases in the abdominal cavity. The patient underwent staging surgery and cytoreduction. Furthermore, the multidisciplinary board decided on adjuvant chemotherapy with an FP regimen (fluorouracil plus cisplatin) because a microscopic metastasis was discovered in the peritoneum near the appendix. Next-generation sequencing showed some pathologic mutations of oncogenes/cancer-associated genes, including *CTNNB1* and *TP53*. This is the fourteenth case of SPN-O and the first one to demonstrate the *TP53* pathogenic mutant variant in SPN-O. The patient showed 8 months of disease-free survival until February 2024. Conclusion: The combination of R0 cytoreduction with FOLFIRI chemotherapy appears to be an effective and feasible treatment option.

## 1. Introduction

Solid pseudopapillary neoplasms (SPNs) are known as a group of rare tumors with low malignant potential, mainly originating from the pancreas (SPN-P) [1,2]. They represent 1–3% of all pancreatic tumors and are rarely detected outside the pancreas [1,2]. Primary SPNs originating from the ovary (SPN-O) are extremely rare [3,4], with only 13 cases reported in the English literature [2,3,4,5,6,7,8,9,10,11,12].

The tumorigenesis of SPNs appears mainly as a result of a mutation in *CTNNB1*, which causes aberrations in the Wnt/β–catenin signaling pathway [6,13]. With advances in molecular studies, a few investigators have identified point mutations in exon 3 of *CTNNB1*, which encodes β-catenin [5,6]. These mutations can lead to oncogenic changes in cell cycle regulation, including cell proliferation, differentiation, or embryonic development [6,14,15].

Here, we report a case of SPN-O to share our experience in managing this extremely rare disease.

## 2. Case Report

**Figure 1:**
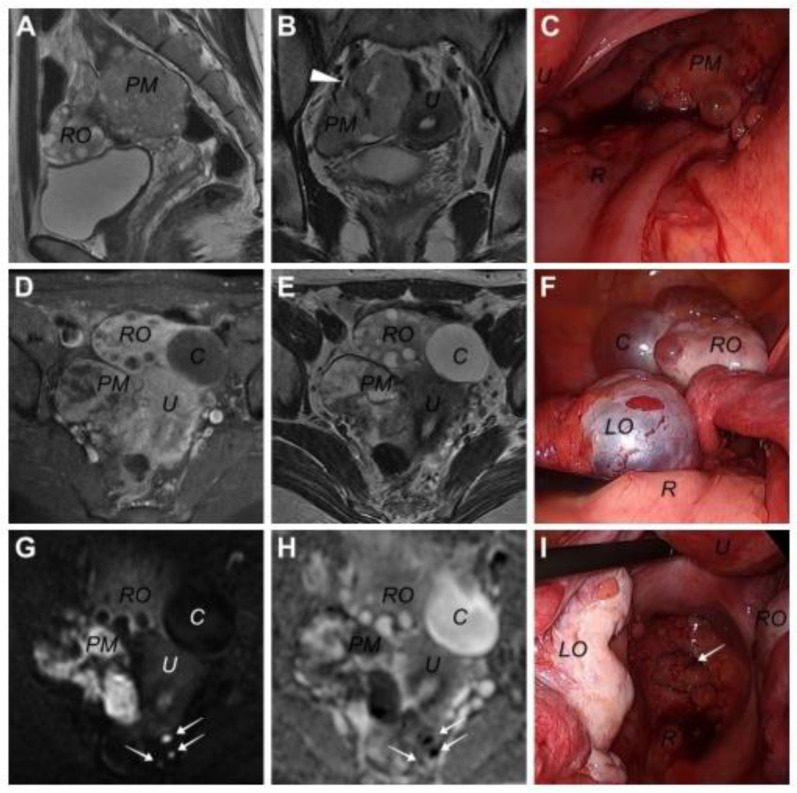
Images of pelvic magnetic resonance imaging and laparoscopic findings in a 31-year-old woman diagnosed with primary solid pseudopapillary neoplasm (SPN) in this case. (**A**) Sagittal T2-weight image, (**B**) coronary T2-weighted image, (**C**) pelvic mass in laparoscopy, (**D**) contrast-enhanced T1-weighted image, (**E**) transverse T2-weighted image, (**F**) both ovaries in laparoscopy, (**G**) diffusion-weighted image, (**H**) the ADC map, and (**I**) metastatic lesions of the peritoneum in laparoscopy. (*PM*: pelvic mass, *RO*: right ovary, *U*: uterus, *R*: rectum, *C*: right ovarian cystic lesion, *LO*: left ovary, arrowhead: right internal iliac artery supplying the pelvic mass, white arrows: metastatic lesions on rectal serosa or peritoneum).

On 10 February 2023, a 31-year-old woman visited our institution with lower abdominal pain. The patient underwent laparoscopic unilateral ovarian cystectomy in another medical institution due to an unknown pathologic diagnosis approximately 4.5 years before the presentation. Whether surgical spillage occurred or not in this laparoscopic surgery was unclear. Computed tomography (CT) and subsequent magnetic resonance imaging (MRI) revealed a 6.5 × 4.0 cm multi-loculated cystic mass lesion arising from the right pelvic wall, along with a 4 cm sized cystic tumor in the right ovary (Figure 1). The pelvic mass was observed to be supplied by the right internal iliac artery in the coronary view of T2-weighted MRI (Figure 1B). Contrast-enhanced T1-weighted MRI revealed contrast enhancement throughout the pelvic mass, except for the internal cystic component (Figure 1D). The pelvic mass exhibited a high signal intensity (SI) on the diffusion-weighted MR image (b = 1000 s/mm^2^), and the apparent diffusion coefficient map showed a signal loss, indicating a highly cellular tumor (Figure 1G). Additionally, some lesions with similar SIs were observed in the rectal serosa, which were later confirmed as tumor seeding in laparoscopic exploration (Figure 1G,H). For this pelvic mass, a neurogenic tumor, such as a benign schwannoma, was suspected, whereas a benign cystic tumor and functional cyst were suspected in the right and left ovaries, respectively. No other abnormal findings, including ascites or lymphadenopathies, and the pancreas, were observed on MRI and CT. This study was approved by the Institutional Review Board of Kyungpook National University Hospital (KNUH 2023-06-015).

On 25 April 2023, the colorectal surgeon in our institution performed laparoscopic exploration. Intraoperatively, the surgeon detected seeding lesions on the rectal serosa and an isolated pelvic mass on the right side of the pelvic cavity. Moreover, the surgeon noted several small cystic or polypoid spherical mass lesions on the surfaces of both ovaries, fallopian tubes, and pelvic peritoneum (see Figure 1C, Figure 1F and Figure 1I, respectively). The mass was laparoscopically removed; subsequently, the surgeon called the gynecologic surgeon to examine the bilateral ovarian lesions. The capsules of the bilateral cystic lesions were resected and sent to a pathologist for frozen section analysis, which revealed the possibility of malignancy. Following permanent pathologic examination, both ovarian capsules and peritoneal lesions were diagnosed with solid pseudopapillary tumors. Following this diagnosis, whole-body positron emission tomography/CT was performed; however, no specific lesion in the pancreas or other distant organs was observed. 

On 15 May 2023, the gynecologic surgeon performed laparotomic staging surgery and cytoreduction, including total hysterectomy, bilateral salpingo-oophorectomy, lymphadenectomy from both pelvic sides up to the para-aortic level, infracolic omentectomy, appendectomy, and multiple peritoneal biopsies. During this surgery, the colorectal surgeon also detected multiple nodular lesions in the anterior side of the rectosigmoid and upper rectum (the lower margin was anterior peritoneal reflection). The surgeon planned en bloc resection with rectal preservation; therefore, to prevent rectal perforation, sharp dissection was performed. Multiple nodular lesions were completely removed, and a rectal tube was placed to prevent perforation and stricture. No residual lesions were macroscopically visible in the abdominal cavity postoperatively. The permanent pathologic examination confirmed the presence of SPN in both ovaries, paragutter peritoneum above the pelvic brim, cecal serosa, and appendix. However, no tumors were noted in the uterus, omentum, pelvis, and para-aortic lymph nodes. Interestingly, despite the absence of any specific macroscopic lesions, microscopic examination revealed metastatic SPN in the peritoneum near the appendix.

**Figure 2:**
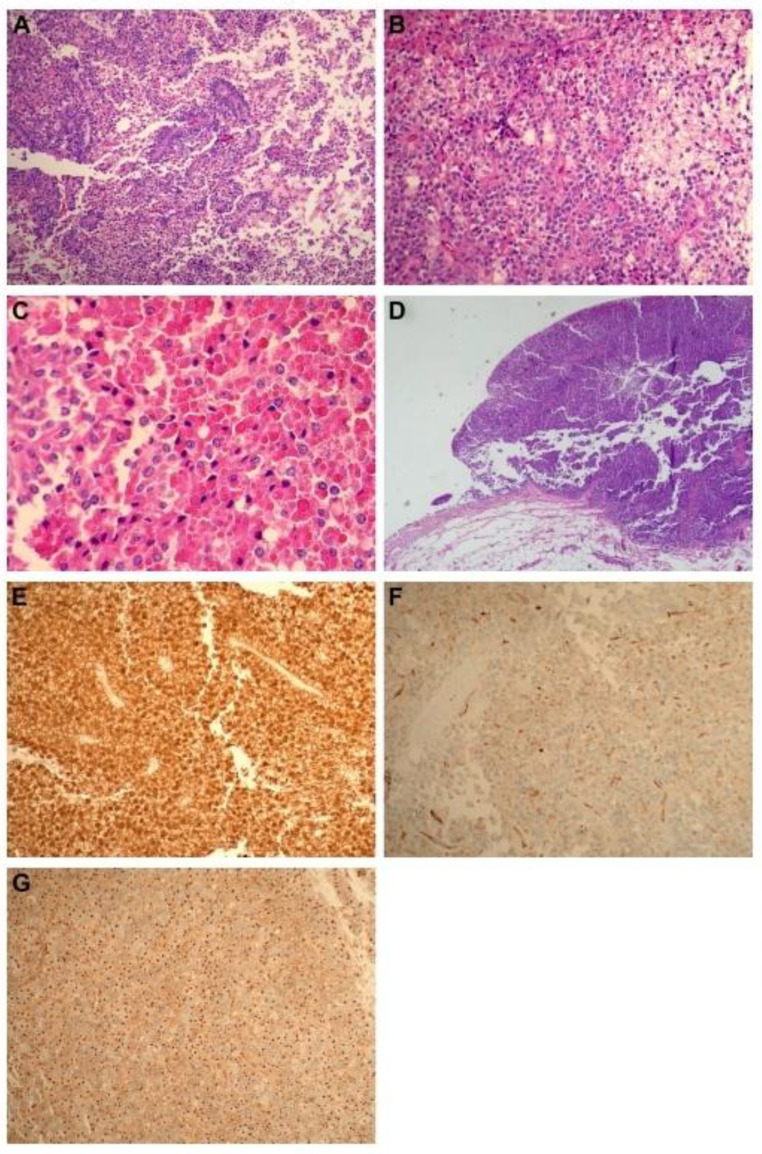
Pathologic findings of a 31-year-old woman diagnosed with primary solid pseudopapillary neoplasm (SPN) in the ovary in this case. (**A**) The tumor shows pseudopapillary architecture due to the discohesion of cells with hyalinized cores (hematoxylin and eosin stain, ×100 magnification). (**B**) Tumor cells are polygonal with eosinophilic-to-pale cytoplasm. Some tumor cells have abundant foamy cytoplasm with paranuclear vacuoles (hematoxylin and eosin stain, ×200 magnification). (**C**) Intracellular and extracellular hyaline globules are frequently observed (hematoxylin and eosin stain, ×400 magnification). (**D**) The peritoneal surface of the right paragut shows a solid tumor deposit (hematoxylin and eosin stain, ×40 magnification). (**E**) The antibodies for β-catenin protein show an abnormal nuclear translocation. (**F**) Immunostaining for WT-1 is focally positive. (**G**) CD99 (MIC2) shows paranuclear dot-like positivity.

The pelvic mass was 7.5 × 5.5 × 4.5 cm in size with a well-capsulated ivory-to-light-brown-colored solid and cystic mass. In the pathologic examination, the tumor showed solid nests with a pseudopapillary architecture. The pseudopapillary patterns were caused by the discohesion of tumor cells with hyalinized cores (Figure 2A). Its cells were polygonal with eosinophilic-to-pale cytoplasm, and some cells had abundant foamy cytoplasm with paranuclear vacuoles (Figure 2B). Intracellular and extracellular hyaline globules were frequently observed (Figure 2C). Interestingly, microscopic examination revealed a metastasis in the peritoneum near the right paragut (Figure 2D). Immunohistochemistry revealed that the tumor cells were positive for β-catenin, which is characteristic and fairly specific for SPN (nuclear translocation), WT-1 (focal nuclear), and CD99, which is a helpful finding for SPN (MIC2; paranuclear dot-like pattern) (see Figure 2E, Figure 2F and Figure 2G, respectively); additionally, cytokeratin was focally positive. Among neuroendocrine markers, synaptophysin and chromogranin were negative; however, CD56 was positive. Inhibin, S-100, and Melan-A showed a negative reaction.

We performed genetic testing using formalin-fixed paraffin-embedded samples of the pelvic mass with next-generation sequencing (NGS), which revealed some missense mutations in multiple genes among a total of 344 target genes with ONCOaccuPanelTM (NGeneBio, Seoul, Korea) [16]. Whereas no mutation was noted in the genes of tier I (variants of strong clinical significance), four missense mutations were noted in tier II (variants of potential clinical significance) genes on the basis of the Association for Molecular Pathology guidelines [17], including *CTNNB1* (c.100G > A), *TP53* (c.329G > T), *FGFR4* (c.379G > C) encoding fibroblast growth factor receptor 4, and *NKX2-1* (c.1106C > T) encoding thyroid transcription factor 1. These point mutations resulted in pathogenic changes in amino acid sequences in each gene, Gly34Arg, Arg110Leu, Asp127His, and Ala369Val, respectively. Moreover, NGS revealed twelve mutations in the genes classified as tier III (variants of unknown clinical significance) or IV (benign or likely benign variants); of the twelve mutations, five were single-nucleotide variants/Indel (*ERBB3*, *MET*, *OR4M2*, *RBBP8*, and *XRCC2*, encoding epidermal growth factor receptor 3, hepatocyte growth factor receptor, olfactory receptor 4M2, retinoblastoma-binding protein 8, and X-ray repair cross-complementing protein 2, respectively), and seven were gene fusion (*ARAF-IGF2*, *ARAF-PHF2*, *DIS3L2-ARAF*, *FLT4-RFX2*, *FLT4-SAMD4B*, *JAK3-RAB11FIP4*, and *NOL9-NKX2-2* [ARAF, IGF2, PHF2, DIS3L2, FLT4, RFX2, SAMD4B, JAK3, RAB11FIP4, NOL9, and NKX2-2 encode serine/threonine-protein kinase, insulin-like growth factor 2, plant homeodomain finger protein 2, DIS3 like 3′-5′ exoribonuclease 2, vascular endothelial growth factor receptor 3, regulatory factor X2, sterile alpha motif domain-containing protein 4B, Janus kinase 3, Rab11 family-interacting protein 4, nucleolar protein 9, and NK2 homeobox 2, respectively]).

Although R0 surgery, leaving no visible lesions, was performed, the multidisciplinary tumor board decided to recommend adjuvant chemotherapy because a microscopic metastasis was observed in the peritoneum near the right paragut. The patient received adjuvant chemotherapy with an FP regimen (D1, cisplatin 60 mg/m^2^ intravenously; D1–D3, 5-fluorouracil 825 mg/m^2^ intravenously) in four cycles every 3 weeks since 29 June 2023. The patient underwent chemotherapy without experiencing any specific adverse effects, except for grade 2 nausea/vomiting and grade 1 oral mucositis. The latest chest and abdomen CT revealed there was no evidence of disease on 8 February 2024.

## 3. Discussion

The features of the ovarian tumor in this case were compatible with the diagnosis of SPN in terms of genetics, histology, and immunohistochemistry. Abdominal CT, pelvic MRI, and laparoscopic findings revealed no specific lesions, indicating that the disease originated from other distant organs. Thus, we believe that this tumor is SPN-O with an advanced stage. We could demonstrate pathogenic mutations in some cancer-associated genes, including *CTNNB1*. Additionally, we first noted a pathogenic mutation in *TP53* in SPN-O.

The patient in this case was shown to have some pathogenic missense mutations in tier II oncogenes/cancer-associated genes through the genetic study with NGS, including *CTNNB1*, *TP53*, *FGFR4*, and *NKX2-1*. Among these mutations, the point mutation of *CTNNB1* (c.100G > A) was in exon 3, similar to a previous study [18]. In the present case, intraperitoneal seeding lesions were detected in the rectum, rectosigmoid, and peritoneum, whereas most conventional SPN cases show low malignant potential. In this case, the advanced disease stage may have resulted from the surgical spillage during the previous surgery, laparoscopic ovarian cystectomy, or pathogenic mutation in *TP53*. Most of the genetic alterations in SPN have been reported in the disease primarily originating from the pancreas. According to a recent study, the *TP53* mutation accompanied by the *CTNNB1* mutation was observed in just a minority of the entire SPN-P group, which seems to have insufficient evidence of correlation with malignant potential or invasiveness in SPNs to date [13]. Additionally, no study has elucidated the aggressive behavior of SPNs on the basis of certain genetic changes. Another recent study on endometrial cancer may provide a clinical implication of mutants in *CTNNB1* and *TP53* correlated with prognosis [19]. The researchers demonstrated a significantly worse recurrence-free survival in a cohort carrying a mutant variant in *CTNNB1* or *TP53* compared with the other cohort without mutations in both *CTNNB1* and *TP53*. Although this result cannot be applied in SPNs owing to the limitation of this study, a similar study can be conducted in SPNs to explain the clinical course or invasiveness of the disease.

Available data on the use of systemic treatment for palliation or adjuvant treatment in SPNs are limited. Furthermore, no standard chemotherapy guidelines have been established. Several clinical cases have proposed the application of diverse chemotherapy, including FOLFOX (oxaliplatin, leucovorin, and 5-fluorouracil) or FOLFIRI (irinotecan, leucovorin, and 5-fluorouracil), or other chemotherapeutic medicines, including gemcitabine, cisplatin, and etoposide. A previous study by Soloni et al. demonstrated that cisplatin, 5-fluorouracil, and gemcitabine were the most commonly administered chemotherapeutic agents for the management of unresectable SPNs [20].

Regarding metastatic disease, our case is similar to the one reported by Syriac et al. [8]. In that study, the patient underwent total abdominal hysterectomy and left salpingo-oophorectomy due to cervical dysplasia 8 years before presentation. The patient appeared to have metastatic lesions in the liver and omentum as well as a left ovarian mass. Whether surgical spillage occurred during the previous surgery and whether the left adnexa had SPN-O were unclear. The authors used adjuvant chemotherapy with a platinum-based drug, paclitaxel and carboplatin. They later switched the drugs to gemcitabine and carboplatin owing to suspected progressive disease in the vaginal apex. The patient received six cycles of chemotherapy but succumbed to the disease 8 months following the initial diagnosis. Kushner et al. recently reported another similar case [2]. In their case, intraoperative spillage occurred during a previous surgery before the patient was referred to their institution. The surgery was robot-assisted laparoscopic hysterectomy and bilateral salpingo-oophorectomy performed owing to a large left adnexal mass. The tumor was diagnosed as SPN-O, and the patient developed multiple intraperitoneal metastases 1 year postoperatively. The patient was considered inoperable, and the authors decided to administer palliative chemotherapy with FOLFIRINOX (5-fluorouracil, leucovorin, irinotecan, and oxaliplatin). However, the patient died from disease-related complications 2 months following the initial visit. In contrast to these two cases, the patient reported by Gahlot et al. managed to survive for over 18 months despite having multiple metastatic lesions in the omentum, pelvic peritoneum, and uterus [3]. The patient underwent surgery without residual lesions, including right salpingo-oophorectomy, total omentectomy, debulking of the tumor, and pelvic lymphadenectomy. Since the surgery, the patient showed no evidence of disease without receiving adjuvant chemotherapy. In our case, owing to the microscopic peritoneal metastasis detected near the appendix, it was decided that the patient would undergo adjuvant chemotherapy with an FP regimen; however, the standard chemotherapy guideline or its benefit has not been established.

## 4. Disease Review

Table 1 shows clinicopathologic features of primary SPN-O cases that have been reported in the English literature. We could find thirteen available cases through PubMed or Google Scholar. In SPN-O, the standard treatment or chemotherapy regimen has not been established due to its rarity.

In molecular terms, the tumorigenesis of SPNs in the pancreas generally resulted from the mutation of *CTNNB1*, which encodes β-catenin. If a mutation occurs in the exon 3 of *CTNNB1*, β-catenin phosphorylation can be disrupted, which is mediated by glycogen synthase kinase-3β. Consequently, the ubiquitin-mediated degradation of β-catenin is inhibited. This aberration results in the accumulation and translocation of β-catenin into the nucleus. The target genes of β-catenin, involved in cell cycle regulation, are stimulated by the nuclear-localized β-catenin, ultimately leading to cell proliferation or differentiation [14,15]. This pathway has also been observed to be the same in SPN-O [5,6]. Therefore, β-catenin immunohistochemistry is considered a robust marker in SPNs. Additionally, E-cadherin is another reliable marker for SPNs. This protein is a calcium-dependent transmembrane glycoprotein which binds with β- or γ-catenin. Since E-cadherin has both extracellular and cytoplasmic domains, it can exhibit different staining features: a lack of staining with an antibody that recognizes the extracellular domain and nuclear positivity with an antibody that recognizes the cytoplasmic domain [6,21,22].

Regarding embryonic development, there is a postulation explaining the tumorigenesis of SPN-O, which involves the transfer of pancreatic tissues to the ovary during development [9]. According to this theory, the left gonad is closely adjacent to the pancreas at approximately 5–8 weeks of gestation, and pancreatic cells may migrate to the gonad during this time [9]. Primary extra-pancreatic sites of SPNs include the mesocolon, liver, omentum, retroperitoneum, paratesticular region, testis, and ovary [2,5,23,24]. Considering the insignificant predominance of SPN-O over testicular SPNs, which are two organs derived from the gonad, the influence of physical distance may be more significant than other factors, including sex hormones [2,25].

According to a recent study, not all SPNs are a result of pathogenic mutations in *CTNNB1* despite the extremely low ratio [13]. Approximately 36% of SPN-P cases were observed to have only a *CTNNB1* mutation; therefore, it generally contains other mutated genes involved in DNA repair pathways [13]. Based on the co-occurring mutated genes, some drugs can be suggested as potential therapies; for example, everolimus showed great efficacy in patients carrying two different pathogenic mutations in *CTNNB1* and *PTEN* [13]. In another recent study, some researchers suggested that *BAP1* and *KDM6A*, encoding BRCA1 associated protein 1 and lysine demethylase 6A, respectively, alterations are molecular markers for the progression of malignancy in SPNs [26]. The patient in this study was revealed to carry no pathogenic variants of these two genes.

Not only were the pivotal genetic alterations not elucidated, but also nor were the reliable prognostic factors. According to a recent study, disseminated disease is not frequently a negative predictor of prognosis [2]. To discover molecular markers or prognostic factors influencing the clinical course of SPNs, further studies are needed.

## 5. Conclusions

Here, we report a case of SPN-O to share our experience in managing this extremely rare disease. We demonstrated a point mutation in exon 3 of *CTNNB1* as a typical case of SPN; this case report is the first to demonstrate a pathogenic mutation in *TP53* in SPN-O. Additionally, we discovered some microscopic metastatic lesions during staging surgery that had not been macroscopically visible. We believe that these findings can provide inspiration for this extremely rare disease. The combination of R0 cytoreduction with FOLFIRI chemotherapy appears to be an effective and feasible treatment option.

## Figures and Tables

**Table 1:** Clinicopathologic features of primary solid pseudopapillary neoplasms of the ovary as reported in the English literature.

Author	Age (Years)	Size * (cm)	Metastasis	*CTNNB1* Mutation	Previous Surgical History	Surgery	Chemotherapy	Treatment Outcome	Length of Follow-Up
Deshpande et al., 2010 [7]	17	25.5				USO, LN		NED	>6 years
	57	3				BSO			
	21	14				USO			
Cheuk et al., 2011 [11]	25	16.5				UO		NED	12 years
Syriac et al., 2012 [8]	45	8	Liver, omentum		Cholecystectomy Appendectomy Gastric bypass TH, USO ^†^	USO, omentectomy, LN, debulking	Paclitaxel and carboplatin to gemcitabine and carboplatin	DOD	8 months
Stoll et al., 2012 [9]	48	8				USO		NED	9 months
Kominami et al., 2014 [6]	18	10		c.110C > T (Ser37Phe)		USO			
He et al., 2015 [10]	39	6				USO		NED	3 years
Gahlot et al., 2016 [3]	25	12	Omentum, uterus, peritoneum			USO, omentectomy, LN, debulking		NED	18 months
Singh et al., 2017 [5]	49	3.8		c.98C > G (Ser33Cys)		TH, BSO		NED	12 months
Komforti et al., 2018 [4]	18	17.5				USO		NED	3 months
Nguyen et al., 2020 [12]	24	5.5				USO			
Kushner et al., 2020 [2]	40 s		Spleen, liver, bowels, vaginal cuff, LNs		Gastric bypass TH, BSO ^‡^	Unresectable	FOLFIRINOX (fluorouracil, leucovorin, irinotecan, oxaliplatin)	DOD	2 months
Current case	31	6.5	Peritoneum, bowels	c.100G > A (Gly34Arg)	UOC	TH, BSO, omentectomy, LN, debulking	FOLFIRI (fluorouracil, leucovorin, irinotecan)	NED	8 months ^§^

Abbreviations: USO, unilateral salpingo-oophorectomy; LN, lymphadenectomy; BSO: bilateral salpingo-oophorectomy; NED: no evidence of disease; DOD: died of disease; TH: total hysterectomy; UOC: unilateral ovarian cystectomy. *: the largest diameter on the image, CT or MRI. ^†^: the diagnosis of the unilateral adnexa is not shown in the article. ^‡^: the diagnosis of the left adnexa is a solid pseudopapillary neoplasm. ^§^: since the staging surgery, including TH, BSO, LN up to the infrarenal level, and cytoreduction.

## Data Availability

Due to patient privacy concerns, the data used in this study are not publicly available. The datasets used during the current study are available from the corresponding author on reasonable request.
